# Flexible Meta-Regression to Assess the Shape of the Benzene–Leukemia Exposure–Response Curve

**DOI:** 10.1289/ehp.0901127

**Published:** 2009-11-18

**Authors:** Jelle Vlaanderen, Lützen Portengen, Nathaniel Rothman, Qing Lan, Hans Kromhout, Roel Vermeulen

**Affiliations:** 1 Institute for Risk Assessment Sciences, Utrecht University, Utrecht, the Netherlands; 2 Division of Cancer Epidemiology and Genetics, National Cancer Institute, Department of Health and Human Services, National Institutes of Health, Bethesda, Maryland, USA

**Keywords:** benzene, epidemiology, leukemia, meta-regression, quantitative risk assessment

## Abstract

**Background:**

Previous evaluations of the shape of the benzene–leukemia exposure–response curve (ERC) were based on a single set or on small sets of human occupational studies. Integrating evidence from all available studies that are of sufficient quality combined with flexible meta-regression models is likely to provide better insight into the functional relation between benzene exposure and risk of leukemia.

**Objectives:**

We used natural splines in a flexible meta-regression method to assess the shape of the benzene–leukemia ERC.

**Methods:**

We fitted meta-regression models to 30 aggregated risk estimates extracted from nine human observational studies and performed sensitivity analyses to assess the impact of *a priori* assessed study characteristics on the predicted ERC.

**Results:**

The natural spline showed a supralinear shape at cumulative exposures less than 100 ppm-years, although this model fitted the data only marginally better than a linear model (*p* = 0.06). Stratification based on study design and jackknifing indicated that the cohort studies had a considerable impact on the shape of the ERC at high exposure levels (> 100 ppm-years) but that predicted risks for the low exposure range (< 50 ppm-years) were robust.

**Conclusions:**

Although limited by the small number of studies and the large heterogeneity between studies, the inclusion of all studies of sufficient quality combined with a flexible meta-regression method provides the most comprehensive evaluation of the benzene–leukemia ERC to date. The natural spline based on all data indicates a significantly increased risk of leukemia [relative risk (RR) = 1.14; 95% confidence interval (CI), 1.04–1.26] at an exposure level as low as 10 ppm-years.

Although broad consensus exists in the scientific community that benzene is a leukemogen, there is considerable uncertainty regarding the actual shape of the exposure–response curve (ERC) (Crump 1996; Paustenbach et al. 1993). Most of the current epidemiologic evidence for an increased leukemia risk stems from studies among workers exposed to relatively high levels of benzene [McDonald 2001; U.S. Environmental Protection Agency (EPA) 1998]. The risks of leukemia at lower benzene exposures, however, remain largely unclear. Importantly, exposure to benzene in occupational settings has dropped considerably during the last three decades (Capleton and Levy 2005). Furthermore, a large proportion of the general population is exposed to low levels of benzene (< 0.2 ppm) through car exhaust, cigarette smoke, and other sources (Johnson et al. 2007). Therefore, the primary interest of current benzene risk assessment is in risks associated with exposure to low levels of benzene.

In existing risk assessments of benzene, the ERC was assessed based on evidence from one “best” study or, alternatively, from a limited set of “best” studies (McDonald 2001; U.S. EPA 1998). These investigations were mostly conducted among relatively highly exposed workers, so the derived ERC might not be directly applicable to workers exposed to lower levels or to the general population. Furthermore, these risk assessments used linear models to describe the ERC, but increasing evidence from molecular epidemiologic studies of workers exposed to a wide range of benzene levels indicates that the shape of the ERC for benzene and its toxic effects may be nonlinear. This hypothesis is based on the observation that the dose-related production of urinary metabolites of benzene, which include the toxic metabolites muconic acid and hydroquinone and the less toxic metabolites phenol and catechol, decreases with increasing benzene exposure (Kim et al. 2006a, 2006b; Rothman et al. 1998). Furthermore, some evidence shows that benzene metabolism favors the production of the toxic metabolites hydroquinone and muconic acid at low exposures (Kim et al. 2006a). This is especially important because hydroquinone is the precursor of 1,4-benzoquinone, which is generally regarded as the most hematotoxic metabolite of benzene (Kim et al. 2006a). The nonlinear production of benzene’s toxic metabolites would have important consequences for risk assessment because one would expect this to result in a nonlinear relationship between benzene exposure and health outcomes as well. Indeed, in a study that looked at the shape of the dose–response curve of benzene-related hematologic effects, a sharper drop in the peripheral white blood cell count was observed at lower levels of exposure (< 1 ppm) than at higher levels (Lan et al. 2004, 2006).

To explore the shape of the benzene–leukemia ERC, we performed flexible meta-regressions on a set of studies that reported results from quantitative exposure–response analysis or benzene and leukemia. For our analyses, we used a modified version of the approach proposed by Bagnardi et al. (2004) that was applied to studies on alcohol and mortality and on silica and lung cancer (Lacasse et al. 2009). It consists of fitting a set of regression models that includes (flexible) regression splines and linear models to aggregated data, adjusting for the expected correlation of estimated (relative) risks within studies. An improvement of this approach over existing meta-regression methods is that the use of regression splines eliminates the need to make strict a priori assumptions regarding the shape of the ERC, which allows for a more objective evaluation of its actual shape.

## Materials and Methods

### Identification of studies and evaluation of study quality

Publications eligible for the meta-regression were identified by a PubMed search that included the MESH key words “benzene,” “humans,” and “leukemia” in combination with either “cohort studies” or “case–control studies.” Other publications were added by following references included in a literature review by Schnatter et al. (2005) that was identified in the original PubMed search and in regulatory risk assessments by the Canadian Centre for Occupational Health and Safety, the U.S. National Institute for Occupational Safety and Health, the U.S. Agency for Toxic Substances and Disease Registry, and the U.S. EPA (Agency for Toxic Substances and Disease Registry 2007; U.S. National Institute for Occupational Safety and Health 1976; Penney 1995; U.S. EPA 1998). The quality of the 11 studies that reported results from quantitative exposure–response analysis for benzene and leukemia (mortality or incidence) was evaluated using a previously developed evaluation framework (Vlaanderen et al. 2008). The first tier of the framework consisted of six criteria that are related to crucial aspects of the quality of the design, the quality of conduct, and the quality of the reporting of human observational studies [see Supplemental Material (doi:10.1289/ehp.0901127)]. A study was excluded from the meta-regression if it did not meet all of the six criteria. Nine studies were of sufficient quality to be included in the meta-regression (Table 1). Two studies that reported results from quantitative exposure–response analysis were excluded: One expressed exposure in undefined units (Guénel et al. 2002), and the other provided insufficient details that resulted in a lack of insight regarding the decisions made in the statistical analysis (Collins et al. 2003).

### Extraction of data from the incorporated studies

A database was constructed based on published data available for the studies incorporated in the meta-regression. We extracted only risk estimates reported for cumulative exposure to benzene (expressed in ppm-years or ppm-months). The database contained the following fields: study identifier, study design, exposure category, risk estimate, confidence interval for the risk estimate, and number of cases and controls for each exposure category (nested case–control studies) or the size of the study population for the exposure category (cohort studies). Three different epidemiologic study designs contributed to the current meta-regression: the nested case–control design (*n* = 3), the cohort design with an internal reference population (*n* = 1), and the cohort design with an external reference population (*n* = 5; Table 1). Reported odds ratios (ORs), relative risks (RRs), and standardized mortality ratios (SMRs) were combined and interpreted as estimates of the RR for the purpose of this meta-regression (McElvenny et al. 2004). The studies selected for the meta-regression were also different with regard to the definition of the reference population that was used. The cohort studies assumed “background (environmental) exposure” in their reference populations (Bloemen et al. 2004; Costantini et al. 2003; Hayes et al. 1997; Rinsky et al. 2002; Swaen et al. 2005; Wong 1987). Typical daily environmental exposure to benzene can range up to 0.2 ppm, which, over a 70-year life span, accumulates to a maximum of 14 ppm-years of cumulative exposure (Johnson et al. 2007). In the nested case–control studies, individuals in the lowest exposure category were used as the reference population (ranging from < 0.17 ppm-years to < 1 ppm-years occupational exposure) (Glass et al. 2003; Rushton and Romaniuk 1997; Schnatter et al. 1996).

### Preparation of the data extracted from the publications

Three steps were necessary to prepare the extracted data for use in the meta-regression models. In the first step, we assigned a specific cumulative exposure estimate to each risk estimate. It is common practice to report only the boundaries of the exposure categories used in an exposure–response analysis, and this was the case for all included studies except the Swaen study, which reported an average mean exposure (Greenland and Longnecker 1992; Swaen et al. 2005). To estimate the mean exposure for the assigned exposure categories, we assumed a log-normal distribution for the cumulative exposure for each study. Any data providing information on the exposure distribution within a study were collected from the publication (i.e., the number of person-years per exposure category, the number of controls per exposure category, or the number of expected cases per exposure category). Maximum likelihood estimation was used to fit a probability density function (PDF) to the available data. Using the PDF, we assigned an average cumulative exposure to each exposure category based on its respective boundaries. To avoid unreasonably high estimates of the average exposure in the highest exposure group, we truncated the exposure distribution at the maximum reported cumulative exposure level. This method of assigning specific cumulative exposures is similar to the approach that was proposed by Hartemink et al. (2006).

In the second step, we estimated the variance of each specific risk estimate to allow weighting based on the precision of the risk estimates in the meta-regression (DerSimonian and Laird 1986). The variance of RRs and ORs was estimated using the reported confidence intervals following a method discussed by Rothman et al. (1982). Estimated variances for studies that reported asymmetrical confidence intervals on the log scale were based on the upper confidence limit only (Boice and McLaughlin 2001; Wartenberg et al. 2000).

In the third step, we estimated the covariance between the different risk estimates within a study by applying the approach advocated by Shi and Copas (2004). This approach is necessary because risk estimates of a study based on a common internal reference group will be correlated. Ignoring this correlation in the meta-regression underestimates the variance of the risk estimates from the study that results in an overestimation of its weight (Bagnardi et al. 2004; Greenland and Longnecker 1992). The Canada-Petrol study (Schnatter et al. 1996) lacked the information necessary to estimate the covariance matrix; therefore, uncorrected variances were used for this study. For studies that reported SMRs, we did not estimate covariance because SMRs within a single study can be assumed to be largely independent when the expected number of deaths used to calculate the SMRs is based on a sufficiently large population.

### Application of the (regression) models to describe the exposure–response relation

Natural spline models (with knots at the 20th, 50th, and 80th percentiles) as well as linear models were fitted to the data to investigate the shape of the exposure–response relation. To improve the statistical properties of the regression models, we fitted all models to the natural logarithm of the reported risk estimates (Berlin et al. 1993). Regression models were fitted to the data using a modified version of a macro developed by Bagnardi et al. (2004). All regression models allowed for (random) study-specific intercepts and exposure effects to accommodate potential between-study heterogeneity (DerSimonian and Laird 1986). Model deviance was used to compare goodness of fit between (nested) models.

### Sensitivity analyses

For two cohort studies Pliofilm (Rinsky et al. 2002) and Dow (Bloemen et al. 2004) multiple updates were available (Table 1). These updates represent multiple reports on the same cohort. In the most recent report, researchers had the opportunity to follow the participants for the longest time since the start of the follow-up period.For these cohorts, the most recent update was included in the meta-regression. To assess the impact of varying follow-up times in our analysis, we also conducted the meta-regression with risk estimates that were abstracted from earlier updates of the Pliofilm and Dow cohorts (Bond et al. 1986; Rinsky et al. 1987). Substitution of the risk estimates did not have a substantial effect on the shape of the predicted ERC or on model fit to the data (data not shown). Sensitivity of the predicted ERC to the inclusion of specific studies was assessed with a jackknifing analysis, excluding one study at a time before (re)predicting the exposure–response relation. In addition, we analyzed the cohort studies (including those with an external reference group as well as the single study with an internal reference group) and nested case–control studies separately and compared their ERC predictions. To allow flexible prediction of the ERC, all sensitivity analyses were done using natural splines.

### Prediction of risk estimates

Benzene–leukemia ERC RRs were estimated for four plausible scenarios at three different levels of cumulative exposure (10, 20, and 40 ppm-years, corresponding to 0.25, 0.5, and 1 ppm intensity of exposure over a tenure of 40 years). We used fitted regression models to predict the risk estimates with associated confidence intervals. In addition, corrected risk estimates and confidence intervals were calculated by subtracting the intercept at zero exposure predicted by the regression model from the risk estimates.

### Software

Average exposure levels for reported exposure categories were estimated using R, version 2.7 (R Core Development Group, Vienna, Austria). All other statistical analyses were performed using SAS software for Windows (version 9.1, SAS Institute Inc., Cary, NC, USA).

## Results

**Figure 1:**
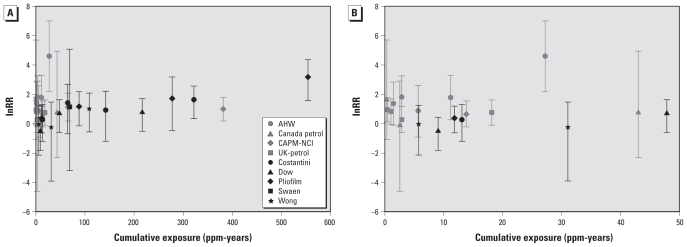
Scatter plot of the risk estimates extracted from the nine studies included in the meta-regression, based on the assigned average cumulative exposure: Full range of cumulative exposures (*A*) and cumulative exposures < 50 ppm-years (*B*). AHW, Australian Health Watch.

Nine studies had sufficient quality to be included in the meta-regression. All included studies were performed in the occupational setting. Together, these studies provided 30 risk estimates over a range of 0.32–554.3 (assigned) ppm-years (Figure 1A). Nineteen (63%) of the risk estimates were assigned a cumulative exposure < 50 ppm-years (Figure 1B). Most of the risk estimates for the lower exposure range were provided by nested case–control studies. The differences in exposure levels between studies can be largely attributed to the different industries in which the studies were performed. The nested case–control studies were all performed in the petroleum industry, whereas the cohort studies were performed in the chemical industry (Pliofilm, Dow, Wong, and Swaen studies), in a shoe factory (Costantini study), or a wide range of different industries [Chinese Academy of Preventive Medicine–National Cancer Institute (CAPM-NCI) study] (Hayes et al. 1997; Table 1).

**Figure 2:**
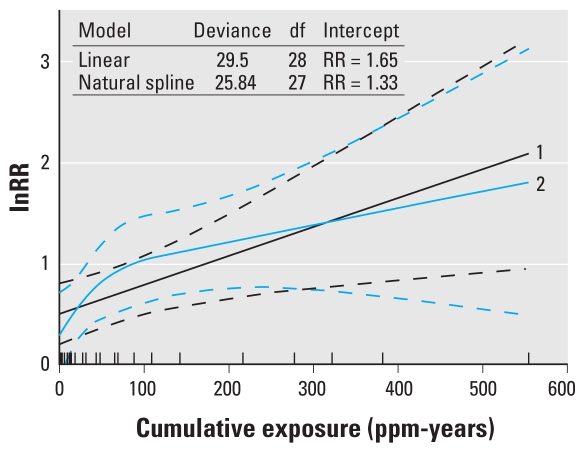
Predicted ERC using all risk estimates from the nine included studies based on a natural spline and linear regression model. Plot 1 is the predicted ERC based on a linear model. Plot 2 is the predicted ERC based on a natural spline model (knots are located at 2.9, 22.7, and 125.5 ppm-years). Dashed lines represent the 95% CIs of the predictions. Rug plot indicates the distribution of the estimated cumulative exposure for each risk estimate included in the analyses.

Predictions of the ERC based on a natural spline model and a linear model are presented in Figure 2. The lower deviance of the natural spline (deviance = 25.84, 27 df) compared with that of the linear model (deviance = 29.25, 28 df) suggests a slightly better fit [chi square test (1 df), *p* = 0.06]. The natural spline model also indicates a strong supralinear shape of the ERC in the low-exposure region, resulting in a considerable lower intercept than the linear model (RR = 1.33 vs. 1.65).

**Figure 3:**
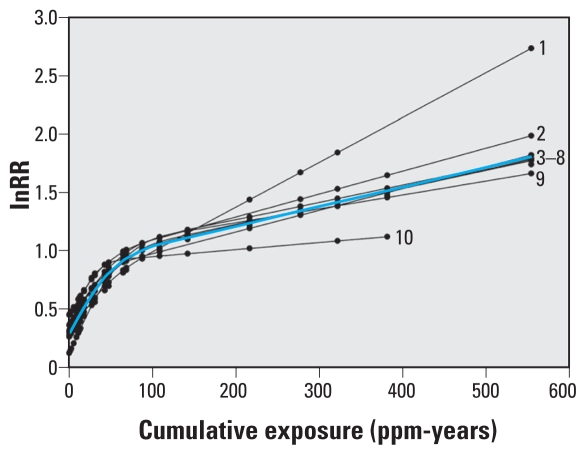
Sensitivity analysis on the prediction of the ERC based on a natural spline. Graph represents nine plots of the predicted ERC based on all studies minus one. The plots are identified by the study that was excluded: 1, CAPM-NCI; 2, Dow; 3, Costantini; 5, US-Chemical (Wong et al. 1987); 6, Swaen; 7, Canada-Petrol; 8, AHW; 9, UK-Petrol; and 10, Pliofilm. Plot 4 is the predicted ERC based on all available studies (blue line). Abbreviation: AHW, Australian Health Watch.

Results from a jackknifing analysis (Figure 3) suggest that the Pliofilm and the CAPM-NCI studies were particularly influential for the (high-exposure region of the) predicted ERC. Exclusion of the Pliofilm study from the meta-regression resulted in a strong reduction of risks predicted for cumulative exposures > 100 ppm-years, whereas exclusion of the CAPM-NCI study had the opposite effect. Exclusion of other studies had little impact on the predicted ERC.

**Figure 4:**
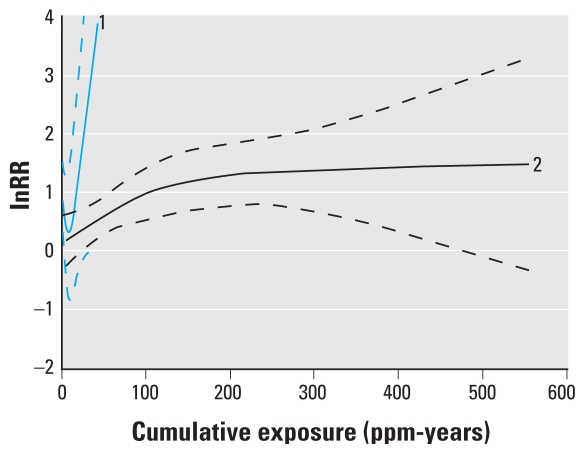
Predicted ERC stratified by study design based on a natural spline. Plot 1 is the predicted ERC based on only the nested case–control studies (knots are located at 1.0, 2.9, and 18.1 ppm-years). Plot 2 is the predicted ERC based on only the cohort studies (knots are located at 13.1, 67.1, and 277.6 ppm-years). Dashed lines represent the 95% CIs of the predictions.

Stratified analyses showed that study design had a considerable impact on the predicted ERC (Figure 4). The ERC based on the cohort studies had a similar shape compared with the ERC based on the full data. However, the supralinear shape was somewhat less pronounced and the predicted intercept slightly lower (RR = 1.13 vs. 1.33). The deviance of the natural spline model fitted to the cohort studies was smaller than the deviance of the corresponding linear model [deviance = 8.43 and 11.97 respectively; chi square test (1 df), *p* = 0.06]. The analysis based on the three nested case–control studies resulted in extremely wide confidence intervals around the predicted ERC and was essentially uninformative (Figure 4).

**Figure 5:**
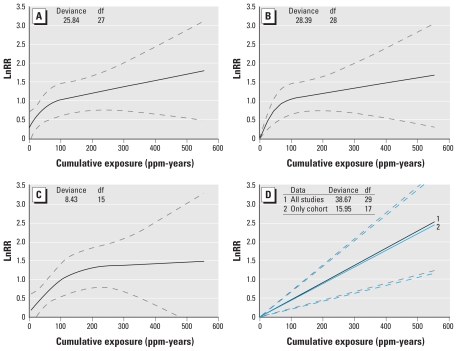
Four different scenarios for the shape of the benzene–leukemia ERC: Natural spline with intercept fitted to all studies (best-fitting model) (*A*), natural spline without intercept fitted to all studies (*B*), natural spline with intercept fitted to the cohort studies (*C*), and linear model without intercept (*D*) fitted to all the studies (1) and only the cohort studies (2). Dashed lines are 95% CIs.

We predicted the RRs for leukemia for three cumulative exposure levels (10, 20, 40 ppm-years) based on four different modeling scenarios for the shape of the benzene leukemia ERC (Table 2). The four scenarios were a natural spline with intercept fitted to all studies (scenario A), a natural spline without intercept fitted to all studies (scenario B), a natural spline with intercept fitted to the cohort studies (scenario C), and a linear model without intercept fitted to all studies (scenario D1) or only the cohort studies (scenario D2; Figure 5). Scenario A predicted the highest RRs (1.52, 1.73, and 2.11 for cumulative exposures of 10, 20, and 40 ppm-years respectively), although these dropped considerably (RR = 1.14, 1.29, and 1.59) after correction for the predicted intercept. RRs predicted in scenario B (RR = 1.22, 1.46, and 1.96) were somewhat lower than the uncorrected RRs from scenario A. Predictions using data from the cohort studies only (scenario C) were also lower than those predicted by scenario A (all studies) (RR = 1.25, 1.38, 1.67), although these differences largely disappeared after we corrected for the intercept. Finally, predictions of the RRs based on scenario D1 (RR = 1.04, 1.09, 1.19) and D2 (RR = 1.05, 1.10, 1.20) were very similar and considerably lower than the predictions based on the other scenarios.

## Discussion

### Interpretation of the predicted ERC

We presented estimates of the benzene–leukemia ERC based on predictions from two regression models. Both the natural spline and linear regression models indicated a positive relation between cumulative exposure to benzene and leukemia risk, although risk appeared to increase more strongly at low exposures in the natural spline model. This supralinear shape of the natural spline model at low exposures is consistent with the increasing evidence that saturable metabolism plays an important role in the low-dose carcinogenicity of benzene (Kim et al. 2006a, 2006b; Lan et al. 2006; Rappaport et al. 2002a, 2002b, 2005b, 2009; Rothman et al. 1998).

Alternative explanations for the nonlinear relation between (inhalatory) benzene exposure and leukemia that we found are depletion of susceptible individuals at high benzene exposure levels and bias due to attenuation of the exposure–response relation within or between studies (Stayner et al. 2003). Attenuation of exposure–response relations is commonly observed in occupational studies and can be caused by several factors, including the healthy worker survivor effect, high disease background rates, exposure measurement error, and confounding and effect modification (Stayner et al. 2003). However, not all of these factors are equally likely to have played a role in occupational studies on benzene and leukemia. Confounding should be considered a potential factor that might have introduced attenuation, but none of the included studies demonstrated (or was able to demonstrate) a confounding effect from potential confounders such as ionizing radiation, smoking, and family history of leukemia (Zeeb and Blettner 1998). In addition, it is unlikely that these factors could have caused serious distortion of the study findings considering the general lack of association with exposure to benzene across the assessed industries (ionizing radiation), weak association with leukemia (smoking), and rare occurrence (family history of leukemia) (Zeeb and Blettner 1998).

Factors that contributed to the heterogeneity observed between studies were differences in study design and exposure assessment. Although all studies were comparable regarding the context of exposure (occupational exposure), considerable differences exist in the geographical location, type of industry, and intensity and frequency of exposure to benzene (Table 1). Differences in study design resulted in different types of risk estimates that were reported: RRs, ORs, and SMRs (Table 1). However, ORs and SMRs can be interpreted as reasonable approximations of the RR when the disease is rare, and these measures have been pooled with RRs for meta-analysis in previous analyses (McElvenny et al. 2004; Steinmaus et al. 2008). According to our evaluation, the quality of exposure assessment was sufficient in all included studies (Vlaanderen et al. 2008). However, systematic differences in exposure assessment strategies between studies might have contributed to the between-study heterogeneity. Because all included studies assessed exposure retrospectively based on a relatively limited set of exposure measurements, exposure estimation in these studies was based partly on decision rules to extrapolate exposure measurements to time periods and exposure circumstances for which no measurements were available (Bloemen et al. 2004; Costantini et al. 2003; Glass et al. 2003; Hayes et al. 1997; Rinsky et al. 2002; Rushton and Romaniuk 1997; Schnatter et al. 1996; Swaen et al. 2005; Wong 1987). The significant amount of expert judgment that goes into these decision rules makes it conceivable that systematic differences in exposure assessment may exist between studies. This situation is illustrated by the exposure assessment for the Pliofilm cohort where three groups of authors have published three different sets of exposure estimates, based on the same exposure measurement data (Crump 1996; Paustenbach et al. 1992; Rinsky et al. 1987, 2002). In contrast, the three nested case–control studies attempted to limit systematic error in exposure assessment by applying similar exposure assessment approaches (Glass et al. 2003; Rushton and Romaniuk 1997; Schnatter et al. 1996).

We tried to assess the potential impact of between-study heterogeneity in the sensitivity analyses. Visual inspection of the results from a jackknifing analysis (Figure 3) showed that two studies that provided risk estimates for the highest assigned cumulative exposures had a considerable impact on the ERC for the higher exposure range. The impact on the lower exposure range was less pronounced. Exclusion of the Australian Health Watch (AHW; Atkinson et al. 2001; Glass et al. 2003) study, which reported relatively high risks for the low-exposure range (Figure 1), had little impact on the shape of the ERC. Results from the sensitivity analysis stratified by study design indicated considerable differences between the ERC based on the nested case–control studies from the petroleum industry and the ERC based on the cohort studies. The shape of the ERC based on the cohort studies only was similar to the shape of the ERC based on all studies. Although the shape of the ERC for the nested case–control studies could be estimated only very imprecisely, the results indicated that these studies were largely responsible for the rather high intercepts that were predicted for the ERCs based on the full data.

Assessing publication bias in a flexible meta-regression is complicated because no standard statistical approaches are available to deal with the correlated effect estimates and the nonlinear exposure–response relations. However, in our opinion it is unlikely that studies with quantitative benzene exposure estimates would not have reported risk estimates for leukemia even if these had been negative because this is one of the major cancer outcomes associated with the exposure. Also, considering the large effort that is required to generate quantitative benzene exposure estimates, it is improbable that any study that had quantitative exposure estimates available would not have been published at all.

Considering that risk estimates in the included studies were calculated in reference to populations with assumed no or negligible occupational exposure to benzene, one might have expected a predicted marginal intercept (ln RR) of approximately 0 (RR = 1) at 0 ppm-years. Although intercepts above 0 are frequently observed in exposure–response studies based on epidemiologic data, most other meta-regression studies have avoided the issue by forcing their regression models to fit through the origin (Bagnardi et al. 2004; Greenland and Longnecker 1992). We did include intercepts in our regression models to attain the best possible fit to the data, which resulted in intercepts of RRs of 1.33 and 1.65 in the natural spline and linear regression models, respectively. An explanation for these nonzero intercepts may be the lack of risk estimates for very low exposure levels (< 0.32 ppm-year). Risk estimates at slightly higher, but still very low, exposure levels (ppm-years) already indicate a strong increase in risk. A natural spline, being linear in its tails, may be unable to track the curvature of the ERC at these low levels, resulting in a nonzero intercept. The same reasoning would apply to intercepts from the linear model, although the effect may be even more extreme. However we cannot exclude the effect of lower-than-expected leukemia risk in the reference populations or (conversely) higher nonbenzene-related leukemia risk in the exposed populations. Finally, attenuation of the ERC due to random and systematic error in the exposure assessment might also have forced the intercept up (Berman and Crump 2008; Stayner et al. 2003).

### Implications of the findings for quantitative risk assessment (QRA)

To facilitate the use of our meta-regression results in QRA, we provided the benzene leukemia ERC for three plausible scenarios (Figure 5, Table 2). In scenario A, the natural spline model was used, which fitted the data slightly better than a linear model. However, the estimate of leukemia risk at 0 ppm-years (the intercept) for this model was much lower than that for the linear model. Because application of these models for risk assessment purposes will most likely entail subtraction of the intercept from all predictions [effectively lowering the predicted (increased) risks for benzene at each exposure level], we favor scenario A (Figure 5A) for risk prediction over the alternatives (Table 2). If one believes that the intercept is due to the natural spline failing to track the shape of the ERC at very low exposures, one may prefer predictions from a spline model without an intercept, thus forcing the predicted ERC through the origin (scenario B, Figure 5B). This model fitted the data only slightly worse than scenario A and may therefore be considered a plausible alternative [chi-square test (1 df), *p* = 0.11]. If one believes that the nested case–control studies should not be used for prediction of the ERC, scenario C (Figure 5C) could be used, which was based on a natural spline model with an intercept fitted to data from the cohort studies only. This scenario resulted in slightly lower predicted risks for exposures < 100 ppm-years. Finally, scenarios D1 (all studies, Figure 5D) and D2 (cohort studies only) were based on linear models without an intercept and are therefore similar in spirit to models commonly used in QRA (McDonald 2001; U.S. EPA 1998) (Figure 5). Clearly, these models fitted the data clearly worse than did the relevant alternative models in scenario A [chi-square test (2 df), *p* = 0.002] and scenario C chi-square test (2 df), *p* = 0.02].

To compare predictions from scenarios A–D with predictions from existing QRAs, we estimated RRs for three cumulative exposures in the low exposure range (10, 20, and 40 ppm-years; Table 2). This showed that risk estimates from our models at these exposures are very similar to those based on the (multiplicative) models used by the U.S. EPA and the California Environmental Protection Agency in their QRA of benzene (McDonald 2001; U.S. EPA 1998). However, although these QRAs were based on data from either the Pliofilm study or the CAPM-NCI study, our approach allowed us to use all available epidemiologic evidence to date and should therefore be more robust. In addition, our approach allowed for a nonlinear shape of the ERC to be used in QRA, which appears to be more appropriate. It is important to note that all analyses were performed on the overarching disease outcome “leukemia.” Slight differences in the definition of this disease existed between studies (Table 1). Unfortunately, analyses for specific subtypes of leukemia will be hampered by a lack of data.

## Conclusion

Flexible meta-regression of the aggregated risk estimates from a set of occupational human observational studies offers an efficient approach to acquiring more insight in the functional relation between exposure to benzene and leukemia. The flexible meta-regression model predicted a supralinear shape of the ERC. Although the limited number of available studies and the large heterogeneity between studies were considerable limitations, sensitivity analyses demonstrated that results were not strongly affected. Our application of a flexible meta-regression method provides the most comprehensive evaluation of the benzene–leukemia ERC to date.

## Figures and Tables

**Table 1:** Details of the studies included in the meta-regression.

Reference (study)	Study design	Risk estimates	Country	Industry	Reference category	Exposure category (ppm-years)		Study outcome	ICD code (revision)^b^	Study population size
						Lowest^a^	Upper			
Wong et al. 1987	Cohort	SMR	USA	Chemical industry	National population death rates	< 15	≥ 60	Mortality	204–207 (8)	7,676 individuals, 6 cases

Hayes et al. 1997 (CAPM-NCI)^c^	Cohort	RR	China	Variety of industries	Workers employed in work units or factories where benzene was not used	< 40	> 100	Incidence	204–208 (9)	74,828 exposed, 35,805 unexposed, 47 cases

Rinksy et al. 2002 (Pliofilm)	Cohort	SMR	USA	Chemical industry	National population death rates	0.01–40	> 400	Mortality	204–208^d^	1,291 individuals, 15 cases

Costantini et al. 2003	Cohort	SMR	Italy	Shoe factory	National and regional specific death rates	< 40	> 200	Mortality	204–207 (8)	1,687 individuals, 11 cases

Bloemen et al. 2004 (Dow)	Cohort	SMR	USA	Chemical industry	National and regional specific death rates	< 28.3	> 79.1	Mortality	204–208 (9)	2,266 individuals, 12 cases

Swaen et al. 2005^e^	Cohort	SMR	Netherlands	Chemical industry	National population death rates	3.4	401.5	Mortality	NA^f^	311 individuals, 1 case

Schnatter et al. 1996 (Canada Petrol)	Nested case–control	OR	Canada	Petroleum industry	Workers exposed to < 0.17 ppm-years	0.18–0.49	8–219.8	Incidence	204–207 (8)	14 cases, 55 controls

Rushton and Romaniuk 1997 (UK-Petrol)	Nested case–control	OR	UK	Petroleum industry	Workers exposed to < 0.26 ppm-years	0.26–0.59	> 4.79	Mortality/incidence	204–208 (9)	90 cases, 354 controls

Atkinson et al. 2001; Glass et al. 2003 (AHW)	Nested case–control	OR	Australia	Petroleum industry	Workers exposed to ≤ 1 ppm-years	1–2	> 16	Incidence	204–208 (9)	33 cases, 165 controls

Abbreviations: AHW, Australian Health Watch; CAPM-NCI, Chinese Academy of Preventive Medicine–National Cancer Institute; NA, not applicable; OR, odds ratio; RR, relative risk; SMR, standardized mortality ratio; UK, United Kingdom.

a Lowest exposure category for which a risk estimate was reported (excluding reference category).

b *International Classification of Diseases* (ICD) used for disease outcomes related to “leukemia”: 204, lymphoid leukemia; 205, myeloid leukemia; 206, monocytic leukemia; 207, other specified leukemia; 208, leukemia of unspecified cell type [World Health Organization (WHO) 1967, 1977].

c Study with internal reference group.

d ICD code for “leukemia” category in effect at time of death of the cases.

e Average mean exposure for tertiles of the exposure distribution; because of a lack of observed cases, a risk estimate was reported only for the middle tertile.

f Disease categorization based on the Dutch electronic file of causes of death.

**Table 2:** Comparison of predicted RRs for three cumulative exposure levels.

			RR (95% CI)		
Model	Deviance (df)	Intercept	10 ppm-years	20 ppm-years	40 ppm-years
Prediction meta-regression—all studies					
Scenario A: natural spline	25.84 (27)	1.33 (0.87–2.05)	1.52 (1.08–2.15)	1.73 (1.27–2.34)	2.11 (1.51–2.96)
Scenario A corrected for intercept			1.14 (1.04–1.26)	1.29 (1.07–1.56)	1.59 (1.15–2.19)
Scenario B: natural spline without intercept	28.39 (28)	NA	1.22 (1.11–1.34)	1.46 (1.22–1.75)	1.96 (1.44–2.68)
Scenario D1: linear model without intercept	38.67 (29)	NA	1.05 (1.02–1.07)	1.10 (1.05–1.15)	1.20 (1.09–1.32)
Prediction meta-regression—cohort studies					
Scenario C: natural spline	8.43 (15)	1.13 (0.71–1.81)	1.25 (0.83–1.88)	1.38 (0.96–1.97)	1.67 (1.22–2.27)
Scenario C corrected for intercept			1.10 (1.04–1.17)	1.22 (1.09–1.36)	1.48 (1.19–1.83)
Scenario D2: linear model without intercept	15.95 (17)	NA	1.04 (1.02–1.07)	1.09 (1.04–1.14)	1.19 (1.09–1.31)

NA, not applicable.
